# Assessment of a Deep Learning Model Based on Electronic Health Record Data to Forecast Clinical Outcomes in Patients With Rheumatoid Arthritis

**DOI:** 10.1001/jamanetworkopen.2019.0606

**Published:** 2019-03-15

**Authors:** Beau Norgeot, Benjamin S. Glicksberg, Laura Trupin, Dmytro Lituiev, Milena Gianfrancesco, Boris Oskotsky, Gabriela Schmajuk, Jinoos Yazdany, Atul J. Butte

**Affiliations:** 1 Bakar Computational Health Sciences Institute, University of California, San Francisco; 2 Division of Rheumatology, Department of Medicine, University of California, San Francisco; 3 San Francisco Veterans Affairs Medical Center, San Francisco, California; 4 Center for Data-Driven Insights and Innovation, University of California Health, Oakland

## Abstract

**Importance:**

Knowing the future condition of a patient would enable a physician to customize current therapeutic options to prevent disease worsening, but predicting that future condition requires sophisticated modeling and information. If artificial intelligence models were capable of forecasting future patient outcomes, they could be used to aid practitioners and patients in prognosticating outcomes or simulating potential outcomes under different treatment scenarios.

**Objective:**

To assess the ability of an artificial intelligence system to prognosticate the state of disease activity of patients with rheumatoid arthritis (RA) at their next clinical visit.

**Design, Setting, and Participants:**

This prognostic study included 820 patients with RA from rheumatology clinics at 2 distinct health care systems with different electronic health record platforms: a university hospital (UH) and a public safety-net hospital (SNH). The UH and SNH had substantially different patient populations and treatment patterns. The UH has records on approximately 1 million total patients starting in January 2012. The UH data for this study were accessed on July 1, 2017. The SNH has records on 65 000 unique individuals starting in January 2013. The SNH data for the study were collected on February 27, 2018.

**Exposures:**

Structured data were extracted from the electronic health record, including exposures (medications), patient demographics, laboratories, and prior measures of disease activity. A longitudinal deep learning model was used to predict disease activity for patients with RA at their next rheumatology clinic visit and to evaluate interhospital performance and model interoperability strategies.

**Main Outcomes and Measures:**

Model performance was quantified using the area under the receiver operating characteristic curve (AUROC). Disease activity in RA was measured using a composite index score.

**Results:**

A total of 578 UH patients (mean [SD] age, 57 [15] years; 477 [82.5%] female; 296 [51.2%] white) and 242 SNH patients (mean [SD] age, 60 [15] years; 195 [80.6%] female; 30 [12.4%] white) were included in the study. Patients at the UH compared with those at the SNH were seen more frequently (median time between visits, 100 vs 180 days) and were more frequently prescribed higher-class medications (biologics) (364 [63.0%] vs 70 [28.9%]). At the UH, the model reached an AUROC of 0.91 (95% CI, 0.86-0.96) in a test cohort of 116 patients. The UH-trained model had an AUROC of 0.74 (95% CI, 0.65-0.83) in the SNH test cohort (n = 117) despite marked differences in the patient populations. In both settings, baseline prediction using each patients’ most recent disease activity score had statistically random performance.

**Conclusions and Relevance:**

The findings suggest that building accurate models to forecast complex disease outcomes using electronic health record data is possible and these models can be shared across hospitals with diverse patient populations.

## Key Points

**Question:**

How accurately can artificial intelligence models prognosticate future patient outcomes for a complex disease, such as rheumatoid arthritis?

**Findings:**

In this prognostic study of 820 patients with rheumatoid arthritis, a longitudinal deep learning model had strong performance in a test cohort of 116 patients, whereas baselines that used each patient’s most recent disease activity score had statistically random performance.

**Meaning:**

The findings suggest that building accurate models to forecast complex disease outcomes using electronic health records is possible.

## Teaser

This prognostic study examines the ability of an artificial intelligence system to predict the state of disease activity in patients with rheumatoid arthritis at their next clinical visit.

## Introduction

Rheumatoid arthritis (RA) is a complex systemic inflammatory disease characterized by joint pain and swelling that affects approximately 1 in 100 people worldwide.^1^ A chronic autoimmune disease, RA is associated with significant morbidity and high costs of care. Disease progression varies greatly among people, and although numerous treatment options exist, individual responses to treatment vary widely.^2^ Although advances in therapeutics and clinical disease management have greatly reduced the proportion of treated patients living with uncontrolled disease activity, remission and durable response are less common. Data from the American College of Rheumatology’s (ACR’s) Rheumatology Informatics System for Effectiveness (RISE) registry indicate that 42% of patients nationwide had moderate or high disease activity at their most recent visit.^3^ These data suggest that additional tools to facilitate and personalize disease management are needed.

Given the volume of data available in electronic health records (EHRs), the number of possible treatment and outcome trajectories associated with heterogeneous patient comorbidities, medications, and other factors is greater than a human, even an experienced physician, can use. Many machine learning methods have been applied to clinical data, such as Cox proportional hazards regression,^4^ random forests,^5^ and LASSO (least absolute shrinkage and selection operator).^6^ However, these methods are often not well suited to forecast outcomes based on EHR data because of the unequal numbers of data points among patients, large amounts of missing data, and highly variable dimensions with time-dependent interactions (eTable 1 and eTable 2 in the Supplement).

Deep learning, a subdiscipline of artificial intelligence, has redefined computer vision^7^ and demonstrated multiple successes in clinical applications^8^ that involve image data for melanoma,^9^ retinopathy,^10^ metastatic breast cancer,^11^ and other biomedical^12^ and health care^13,14^ domains. Deep learning is being applied to a rapidly increasing number of EHR-related data sets,^15^ and like the application of technology to any new field, there are numerous opportunities and challenges.^12,16^ A subfamily of deep learning called recurrent neural networks has become state of the art in longitudinal predictions,^17^ solving complex problems in sequence-modeling fields, such as language translation^18^ and self-driving cars.^19^ Longitudinal deep learning models have previously been applied to EHR data,^20,21^ classifying cardiovascular arrhythmias^22^ and predicting inpatient mortality and emergency department readmissions.^23^ To our knowledge, there has been no attempt to forecast RA disease activity in future clinic visits using any deep or machine learning approach.

In the current study, we aimed to use structured data from the EHR to build a model that would most accurately predict RA disease activity. If successful, the ability to forecast disease activity could be clinically used to inform the aggressiveness of treatment on an individualized basis at each clinical visit. Models developed for predicting RA disease activity may be informative for other health conditions with quantifiable outcomes in the outpatient setting.

## Methods

### Data Sources

Data for this study were extracted from the EHRs of 2 different hospitals: a university hospital (UH) rheumatology clinic (University of California, San Francisco) and a rheumatology clinic from a safety-net hospital (SNH) (Zuckerberg San Francisco General Hospital). The UH uses an Epic EHR system that contains records on approximately 1 million total patients starting in January 2012. The UH data for this study were accessed on July 1, 2017. The SNH uses separate EHR vendors for inpatients and outpatients; eClinicalWorks is used for outpatients, and the EHR contains records on 65 000 unique individuals starting in January 2013. The SNH data for the study were collected on February 27, 2018. A detailed description of the methods of EHR access can be found in the eAppendix in the Supplement. We followed the Transparent Reporting of a Multivariable Prediction Model for Individual Prognosis or Diagnosis (TRIPOD) reporting guideline.^24^ This study was approved by the University of California, San Francisco Committee on Human Research, which issued a research exemption for the deidentified pre-existing clinical data used in this study. Deidentified pre-existing clinical data from Zuckerberg San Francisco General Hospital was obtained under the research protocol 15-18282.

### Definition of the Cohort With RA

Patients had to have 2 RA-related *International Classification of Diseases, Ninth Revision* diagnostic codes (any of 714.0, 714.1, or 714.2) spaced a minimum of 30 days apart as classified by a rheumatologist and been prescribed at least 1 disease-modifying antirheumatic drug (DMARD). These criteria have shown high specificity in an RA cohort study.^25^ To further increase specificity, we required that each patient have a minimum of 2 clinical disease activity index (CDAI) scores that were assigned only by rheumatologists for patients with RA at both clinics in this study. In addition, we required patients to have 1 RA diagnostic laboratory value (either C-reactive protein [CRP] level or erythrocyte sedimentation rate [ESR]). These criteria were considered to verify that included patients were being treated for RA at the clinic for a minimum of 4 months. Final cohort sizes that met inclusion criteria were 578 UH patients and 242 SNH patients.

### RA Disease Outcome Metric

The ACR endorses 6 different disease activity measures. The CDAI, a composite index of patient and physician assessments and scoring of tender and swollen joints, is the most frequently used activity measure in the RISE registry and is the primary score used at both the UH and SNH. The CDAI is recorded as a raw score (range, 0-72) but is subsequently binned into 4 categories: remission (≤2.8), low (2.9-10), moderate (>10 to 22), or high (>22) disease activity.^26,27^ These 4 categories can then be further aggregated into a binary disease activity state: controlled (remission or low activity, CDAI≤10) or uncontrolled (moderate or high activity, CDAI>10).

### Variables Used in the Model

Given the relatively small number of patients available and the complexity of longitudinal models, we chose to include only variables with known clinical significance. These variables are known to be associated with disease activity; however, no study to our knowledge has shown them to be predictive of future disease activity. We included the following variables: prior CDAI score, ESR and CRP level, DMARDs (eTable 3 in the Supplement), oral and injected glucocorticoids, autoantibodies (presence of rheumatoid factor and/or anticyclic citrullinated peptides), and demographics (age, sex, and race/ethnicity). We chose to include only the first occurrence of each medication because of the lack of reliable medication treatment stop dates in the EHR. Considering each variable at each of 4 different time windows resulted in a reasonably large total time-dependent variable space of 165 variables (29 possible DMARDs, 8 possible corticosteroids, CDAI score, ESR, and CRP level at each time window in addition to the 5 static variables: demographics and anticyclic citrullinated peptides and rheumatoid factor).

### Modeling

Data were sorted chronologically by patient. The patient demographics and history of clinical and laboratory variables were used to predict their most recent disease activity. Extensive experimentation was performed to determine the optimal methods to format the chronological data as input and construct and train the most accurate deep learning model for outpatient forecasting within this data set. A fully dense architecture was included during model selection as a surrogate for logistic regression. Complete information pertaining to model input, building, and selection as well as a TRIPOD^24^ checklist are included in the eAppendix in the Supplement. The code to build and train the model is openly available on github.^28^

### Comparative Baselines

There are no clinical protocols, to our knowledge, for predicting a patient’s CDAI score at a future visit. Therefore, as a first baseline, we built a classifier that uses a Bayesian prior on the likelihood of each outcome category (outcome posterior classifier). For example, if the ratio of controlled-to-uncontrolled outcomes was 60:40, as in the case of the UH cohort, the model would assign a forecasting prediction of controlled 60% of the time. As a second baseline (change posterior classifier), we built a classifier with 2 elements of prior knowledge. First was each individual patient’s previously recorded outcome. Second was the likelihood of changing outcome classes from one encounter to the next. We then built a model that considered each patient’s previous outcome class as well as the likelihood within the cohort to switch classes to forecast the patient’s future outcome class. For example, if the probability of switching outcome classes was 30%, the model would look at the previous outcome for each patient, and to forecast the patient’s future condition, it would change the class of 30% of the patients while maintaining the class of the remaining 70% of the patients.

### Evaluation Criteria

We chose the area under the receiver operating characteristic curve (AUROC) as our primary evaluation metric. In addition to AUROC, we performed sensitivity analyses to better evaluate the top model’s potential clinical utility. We assessed how often the model was confident and wrong using a threshold of 0.50 and compared model performance among groups of patients whose CDAI score at the predicted visit was remission, low, moderate, or high disease activity. We explored how forecasting models may be shared across institutions that may not be able to directly share patient data. We evaluated the association of the number of training samples with the models’ performance to estimate the patient cohort size at which a hospital should decide to use a model trained at a different hospital instead of building one of their own.

### Model Explanation and Interpretability

We calculated permutation importance scores (PISs)^29^ to measure the contribution of each independent variable, including time, to the overall model performance measured by AUROC. We generated a confusion plot by collecting the final dense representation learned by the model for each patient and plotting them using t-distributed Stochastic Neighbor Embedding to assess the coherence of the representations learned by the model.

### Performance in a Distinctly Different Cohort

We assessed 3 different methods of using the model among patients from the second health care system (SNH). We selected the top-performing model architecture via Bayesian optimization using the UH training cohort. First, we trained a model using the small SNH training cohort. Second, we tested the model trained on the larger UH training cohort directly on the SNH test cohort. Third, we used model transfer learning and fine-tuning to update the fully trained UH model using the SNH training cohort. An explanation of the theory of transfer learning and fine-tuning as well as the methods that we applied can be found in the eAppendix in the Supplement.

### Statistical Analysis

The AUROC CIs were generated from the UH validation cohort for model selection and from the UH and the SNH test cohorts for final performance assessment using the Delong method.^30^ Models with an AUROC CI that spanned 0.5 are not statistically different from random performance. Variables at each time point for which PIS CIs spanned the baseline AUROC were considered to be insignificant.

## Results

### Clinical Cohort Comparison

A total of 578 UH patients (mean [SD] age, 57 [15] years; 477 [82.5%] female; 296 [51.2%] white) and 242 SNH patients (mean [SD] age, 60 [15] years; 195 [80.6%] female; 30 [12.4%] white) were included in the study (Table). The UH population was seen by rheumatologists with nearly double the frequency (median time between visits, 100 vs 180 days), and a broader spectrum of treatment was used compared with the population at the SNH. The UH patients were more than twice as likely as the SNH patients to be prescribed higher-class medications (364 [63.0%] in the UH cohort and 70 [28.9%] in the SNH cohort were prescribed biologics).

**Table.** Characteristics of Individuals With Rheumatoid Arthritis in the 2 Health Care Systems Studied^a^

Characteristic	University Hospital Cohort (n = 578)	Safety-Net Hospital Cohort (n = 242)
Age, mean (SD), y	57 (15)	60 (15)
Female	477 (82.5)	195 (80.6)
Race/ethnicity		
White	296 (51.2)	30 (12.4)
African American	33 (5.7)	19 (7.9)
Hispanic	97 (16.8)	89 (36.8)
Asian	101 (17.5)	70 (28.9)
Other	51 (8.8)	34 (14.0)
EHR system	Epic	eClinicalWorks
Median No. of CDAI scores per patient	6	4
Time between CDAI, median (range), d	100	180
DMARD^b^		
Conventional synthetic	534 (92.4)	191 (78.9)
Biologic	364 (63.0)	70 (28.9)
Tofacitinib	29 (5.0)	0

Abbreviations: CDAI, clinical disease activity index; DMARD, disease-modifying antirheumatic drug; EHR, electronic health record.

^a^ Data are presented as number (percentage) of patients unless otherwise indicated.

^b^ Number of patients prescribed a DMARD at the clinic before their index date. eTable 3 in the Supplement gives the medications considered for each DMARD category.

### Primary Cohort (UH) Results

**Figure 1.**
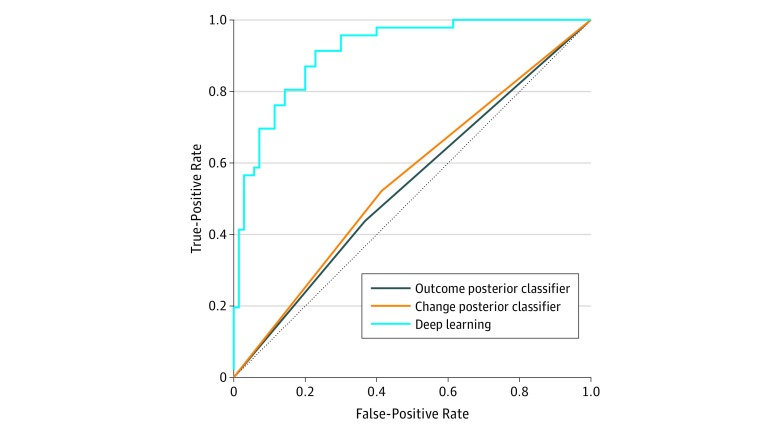
Forecasting Performance of the Deep Learning Models in the University Hospital (UH) Cohort The distribution of outcomes from the training cohort at UH was 60% controlled and 40% uncontrolled according to the clinical disease activity index. This was previously used to train the outcome posterior classifier at UH (area under the receiver operating characteristic curve [AUROC], 0.535). The likelihood of switching outcomes between visits within the training cohort was 25%. This was used previously to train the change posterior classifier at UH (AUROC, 0.554). Deep Learning produced the best results (AUROC, 0.912).

The best-performing model was small and highly regularized and consisted of a time-distributed layer followed by gated recurrent unit layers and a final dense layer (eFigure 1 in the Supplement). Fixed intervals of 120 days; random sampling during training; equal penalization of errors for both classes; use of a combination of clinical, medication, and laboratory variables; and 1 year of each patient’s history before their index date provided the best results. The best deep learning model (Figure 1) demonstrated excellent forecasting performance (AUROC, 0.91; 95% CI, 0.86-0.96) in the UH test cohort (n = 116). Both baselines demonstrated near-random performance (outcome posterior classifier: AUROC, 0.54; 95% CI, 0.44-0.63; change posterior classifier: AUROC, 0.55; 95% CI, 0.46-0.64).

### Sensitivity and Model Explanation

**Figure 2.**
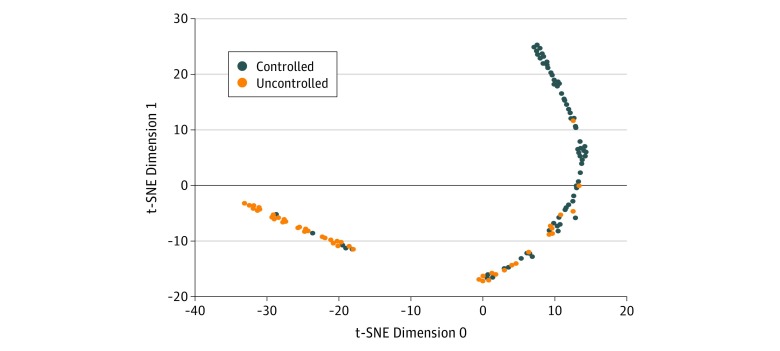
Confusion Plot Confusion plot consisting of the final embedding of the model, the learned patient trajectory vectors, visualized using t-distributed stochastic neighbor embedding, with colors according to the ground truth of the patients outcome at their next visit. The model places observations onto a 1-dimension manifold with controlled and uncontrolled outcomes clustering along different ends of the manifold.

A sensitivity analysis comparing forecasting performance with the number of samples available for training revealed a nonlinear increase in performance with linear increases in sample size (eFigure 2 in the Supplement). The CDAI was important for forecasting performance in each time window (combined PIS, 40) followed by time (PIS, 11). The ESR and CRP variables contributed small but significant predictive power to the 2 most recent time windows (combined PIS was 2 for ESR and 3 for CRP). Corticosteroids as a class at the current time window had a PIS of 4, with prednisone alone having a PIS of 2; these variables were not significant in other windows. Multiple individual DMARDs were significant variables, but each had a PIS less than 2. The model was confident (probability of having uncontrolled disease activity at the next visit, >0.8 or <0.2) and incorrect for only 2 of the 117 test samples (1.7%). These errors occurred for patients whose future visit CDAI score fell on the threshold between the outcome classes (CDAI score range, 8-12). Performance was equal among patients whose future CDAI score was clinically determined as remission, low activity, or high activity. Predictive performance was lowest for patients whose future disease activity was moderate; most of the incorrectly classified patients in this group had CDAI scores near the classification threshold (CDAI score range, 10-14). The confusion plot (Figure 2) appears as a nearly 1-dimensional manifold (a curve). Instead of dichotomous clusters for each outcome category, the model learned a continuous representation of the patients. Distinct decision boundaries based on the confusion plot can be seen.

### Secondary (SNH) Cohort Results

**Figure 3.**
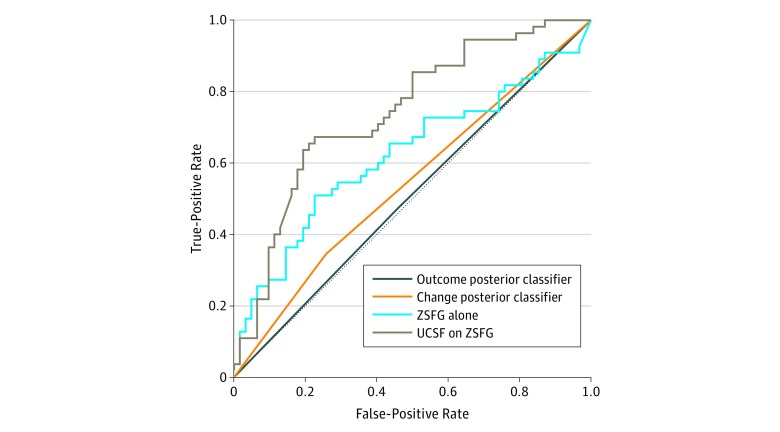
Forecasting Performance of the Deep Learning Models in the Safety-Net Hospital Cohort UCSF indicates University of California, San Francisco; ZSFG, Zuckerberg San Francisco General Hospital.

When the top-performing model architecture was trained on the SNH training cohort (n = 125) (Figure 3), it produced an AUROC of 0.62 (95% CI, 0.52-0.72) in the SNH test cohort (n = 117). Use of a model that was trained on all the UH patients (n = 578) directly in the SNH test cohort was associated with increased forecasting performance (AUROC,  0.74; 95% CI, 0.65-0.83). Use of transfer learning and fine-tuning to update the UH-trained model using the SNH training cohort was not associated with any additional improvements in performance (AUROC, 0.74). Both baselines demonstrated random performance (outcome posterior: AUROC, 0.51; 95% CI, 0.39-0.62; change posterior classifier: AUROC, 0.54; 95% CI, 0.46-0.62).

## Discussion

In this study, we identified the best deep learning approach to forecast future RA disease activity scores across 2 health care systems and compared those results with prediction models that used only a patient’s most recent CDAI score. Contrary to our expectations, a patient’s most recent CDAI score alone was a poor predictor of their index CDAI score, as evidenced by the statistically random results of both baseline predictive models. The combination of history of disease activity, laboratory values, and medications was the strongest predictor of the disease activity at the next visit. More than 20 variables were found to be significantly important for predictive accuracy, which is a relatively small number; however, these variables have time-dependent interactions, which add considerable complexity. For example, our best deep learning model substantially outperformed the multilayer perceptron (which acts equivalently to logistic regression^31^) (eTable 1 in the Supplement), suggesting the utility of more-complex deep learning models for this task.

Our results suggest that deep learning models can be successfully trained on cohorts of only a few hundred patients to accurately predict outcomes in patients with RA by using EHR data. We also found that our model performed well when applied to a second health care system with a distinct sociodemographic population and separate EHR system, although we acknowledge that the performance was poorer compared with the first health system. Given the many differences in the demographics and social determinants among the patients in these centers, we believe that the ability of the model to function significantly above random is still promising. By considering no more than the most recent year of each patient’s history but allowing patients to have as few as 4 months of history, the model may have utility for patients at all stages of their care, if proven in future prospective studies. Although the amount of data that a rheumatologist must synthesize in a single visit to make decisions is large and increasing, the results presented herein suggest that use of artificial intelligence models to assist with predictive tasks in the near future is promising.

Early successes in the application of deep learning to clinical forecasting^22,23,32^ demonstrated that longitudinal deep learning models outperformed traditional machine learning approaches and that reasonable predictive performance was possible. However, these studies^22,23,32^ were limited in their clinical utility by the inclusion of patients without clinical risk indicators for the outcomes being predicted, the numbers of patients used for training, and a lack of evaluation of model performance across hospitals with diverse patient populations. This study addresses these open questions by focusing on a clinically relevant patient population and outcome at a large UH and an associated SNH. The model trained on the larger UH population produced the best results in the SNH cohort, demonstrating the power of larger training sizes and the interoperability of models among hospitals with diverse patient populations. Although use of transfer learning and fine-tuning methods to update the fully trained UH model using the SNH training cohort did not provide any additional improvements in performance, we suspect that this was because the SNH training cohort was too small and thus overfitting occurred.

The future decision support system that we envision will involve aggregating data from multiple institutions, training the model on all those data, and then using the model in small clinics as well as large hospital systems, giving everyone access to the most robust models trained on the largest and most diverse patient populations possible. Use of such a forecasting model may help physicians and patients understand and predict disease trajectories, which in turn could help inform the aggressiveness of treatment. We believe that there are many clinical situations in which there is equipoise about whether and how to augment therapy for RA. Patients may be stable for some time but have a CDAI score over the threshold of moderate disease activity at their clinic visit. Alternately, they may have experienced moderate disease activity during several visits and adverse effects related to their current DMARD regimen. In situations such as these, in which waiting until the next visit to consider any medication changes seems like a reasonable option, having a prediction from the algorithm that indicates that the CDAI score at the next visit will likely be worse may push a practitioner and patient to action. We believe that these marginal situations are the ones most likely to benefit from the algorithm. Given the algorithm’s strong performance at identifying patients predicted to have controlled disease activity at their next visit, probability thresholds could be analyzed to specifically improve outcomes for patients in these marginal situations. As a patient’s health status and other variables change, the model may adapt its predictions, allowing patients and practitioners to use this information to inform treatment changes dynamically. As we move toward personalized medicine, such models can be used to simulate trajectories given different treatment scenarios. The addition of molecular, genomic, and other types of data to EHR data to generate treatment response trajectories would allow a more personalized medicine approach to RA care.

With large national registries, such as the ACR’s RISE registry, now available for rheumatic and other diseases, we see a rich future in the application of deep learning to longitudinal patient care. Model performance is nearing the point at which models are good enough to warrant launching a prospective clinical trial to evaluate their usefulness in aiding practitioners and patients to prognosticate RA outcomes or simulate outcome trajectories under different treatment scenarios.

### Limitations

With data from 2 distinct hospital systems and more than 800 total patients, inferences about the generalizability of models cannot be made. Accordingly, we acknowledge that this study is limited to being a proof-of-concept study. To our knowledge, there are no clinical methods for explicitly forecasting disease activity in patients with RA at future visits, and no method for this has ever been used in a clinic. Although this lack of methods underscores the need for the work introduced in this study, it leaves us without any clinical baseline with which to compare machine learning results.

There are numerous inherent biases in medicine; perhaps most significant are those relating to sicker patients generally having a greater number of data points than healthier patients. We sought to address this bias at multiple levels. We gave all patients the same number of time windows, set to be equal to the smallest median number of visits in either cohort (Table), and then eliminated all but the most recent values within each window for a patient. In addition, including fewer variables in the model was done to reduce the risk of spurious associations among variables. There is still the potential for biases. For example, physicians may choose to order or not order laboratory tests for a given patient at a given visit based on factors that were not modeled in our method, including physician preference. Similarly, some physician-patient combinations may be more or less likely to switch a patient’s treatment strategy.

An associated challenge was the decision about what to do when the value of a patient’s variable was missing from a time window. Statistical imputation or forward filling are likely to introduce or reinforce bias for a health condition that varies greatly among individuals and within a person over time and when most patients’ disease activity is uncontrolled. Our choice to replace missing continuous variables with a value never observed in our data set (zero) was not perfect because the replaced value more closely resembled healthy patients than sick ones, but it seemed to be the replacement option that was least likely to reinforce the dominant bias of sicker patients contributing more data to models. Although we strove to reduce biases in our data and modeling, we could not fully eliminate them. Using the treating physician as the variable to model, which was not possible in this current study, could potentially reduce bias further in the future.

Finally, the performance of the UH-trained model on the SNH test cohort (AUROC, 0.74) was too low to be of immediate clinical utility; however, the performance revealed that the model learned robust and transferable data. Given the notable differences in the 2 clinical populations, a method of exploring whether the differences in model performance between the populations was exclusively attributable to the differences in the treatment populations would have added clarity to the study. Although the patient populations were different in many ways that we can measure (Table), they were also different in many ways that we could not reliably measure (eg, other socioeconomic factors, environment, social structure, and insurance coverage), making an actual patient-matching algorithm impossible given the small sizes of both populations. For complex chronic diseases, such as RA, patient populations in the hundreds are unlikely to capture enough clinical or social variation to adequately represent the complete disease spectrum. Our sensitivity analysis revealed that increasing the training set size was associated with nonlinear increases in model performance. Thus, adding EHR data from other institutions may add to the predictive power and insights into the subtler factors associated with the model’s performance.

## Conclusions

The findings suggest that building accurate models to forecast complex disease outcomes using EHR data is possible and these models can be shared across hospitals with different EHR systems and diverse patient populations. In the future, models built from large pooled patient populations may be the most accurate, giving everyone access to the most robust models trained on the largest and most diverse patient populations possible. The methods used to develop models for predicting RA disease activity may be informative for other health conditions with quantifiable outcomes.

## Notes

Supplement. **eTable 1.** Model Selection Results **eTable 2.** Contrastive Comparison of Machine Learning Methods **eTable 3.** List of Medications Identified for RA Treatment and Considered DMARDs for the Purposes of This Work **eAppendix.** Supplemental Methods and Discussion **eReferences** **eFigure 1.** Architecture of Best Performing Longitudinal Deep Learning Model **eFigure 2.** Sensitivity Analysis Comparing Forecasting Performance versus Training Size
