# Supplementary material for: Assessment of a Deep Learning Model Based on Electronic Health Record Data to Forecast Clinical Outcomes in Patients With Rheumatoid Arthritis
Source: JAMA Netw Open. 2019 Mar 15;2(3):e190606. doi: 10.1001/jamanetworkopen.2019.0606 (PMC6484652; doi:10.1001/jamanetworkopen.2019.0606)
Supplement: Supplement. — eTable 1. Model Selection Results eTable 2. Contrastive Comparison of Machine Learning Methods eTable 3. List of Medications Identified for RA Treatment and Considered DMARDs for the Purposes of This Work eAppendix. Supplemental Methods and Discussion eReferences eFigure 1. Architecture of Best Performing Longitudinal Deep Learning Model eFigure 2. Sensitivity Analysis Comparing Forecasting Performance versus Training Size [file jamanetwopen-2-e190606-s001.pdf]

Supplementary Online Content

Norgeot B, Glicksberg BS, Trupin L, et al. Assessment of a deep learning model based on electronic health record data to forecast clinical outcomes in patients with rheumatoid arthritis. *JAMA Netw Open.* 2019;2(3):e190606. doi:10.1001/jamanetworkopen.2019.0606

eTable 1. Model Selection Results

eTable 2. Contrastive Comparison of Machine Learning Methods

eTable 3. List of Medications Identified for RA Treatment and Considered DMARDs for the Purposes of This Work

eAppendix. Supplemental Methods and Discussion

eReferences

eFigure 1. Architecture of Best Performing Longitudinal Deep Learning Model

eFigure 2. Sensitivity Analysis Comparing Forecasting Performance versus Training Size

This supplementary material has been provided by the authors to give readers additional information about their work.

© 2019 Norgeot B et al. *JAMA Network Open.*

eTable 1. Model Selection Results

Aggregation Function	Dense	GRU	LSTM	CNN	TDD Dense	TDD GRU	TDD LSTM	TDD CNN	TDD Causal CNN
AUC [95% CI]	0.778 [0.683, 0.864]	0.811 [0.730, 0.888]	0.790 [0.695, 0.874]	0.782 [0.675, 0.871]	0.817 [0.731, 0.894]	**0.924 [0.867, 0.974]**	0.917 [0.833, 0.971]	0.896 [0.797, 0.957]	0.912 [0.830, 0.966]

Fully connected networks perform non-randomly but had the lowest overall performance. GRU, LSTM, CNN models perform nearly equivalently. Adding a single dense function applied across all variables within each time point (TDD) produced the largest increase in model performance. The best performing architecture utilized a TDD input layer followed by a recurrent layer with a slight preference for GRUs over LSTMs.

© 2019 Norgeot B et al. *JAMA Network Open.*

eTable 2. Contrastive Comparison of Machine Learning Methods

Table 1: Comparison of machine learning algorithms

ML Method	Models Time	Interactions	Unequal Length inputs	Can handle missing data	Can handle high dimensions	Number of training samples needed	Potential to overfit
Cox	Yes	No	No	No	No	low	low
Random Forest	No	Yes	No	Yes	No	low	low
LASSO	No	No	No	No	Yes	Medium	Medium
RNN	Yes	Yes	Yes	Yes	Yes	High	High

© 2019 Norgeot B et al. *JAMA Network Open.*

eTable 3. List of Medications Identified for RA Treatment and Considered DMARDs for the Purposes of This Work

**Drug Name**	**Class**
Methotrexate	Small Molecule
Sulfasalazine	Small Molecule
Hydroxychloroquine	Small Molecule
Leflunomide	Small Molecule
Azathioprine	Small Molecule
Auranofin	Small Molecule
Chloroquine	Small Molecule
Cyclophosphamide	Small Molecule
Cyclosporine	Small Molecule
Gold	Small Molecule
Minocycline	Small Molecule
Mycophenolate	Small Molecule
Penicillamine	Small Molecule
Myocrisin	Small Molecule
Abatacept	Biologic
Adalimumab	Biologic
Anakinra	Biologic
Certolizumab	Biologic
Etanercept	Biologic
Golimumab	Biologic
Infliximab	Biologic
Rituximab	Biologic
Tocilizumab	Biologic
Inflectra	Bio-Similar
Remsima	Bio-Similar
Benepali	Bio-Similar
Maball	Bio-Similar
Tofacitinib	JAKs Inhibitor

© 2019 Norgeot B et al. *JAMA Network Open.*

eAppendix. Supplemental Methods and Discussion

<u>Data</u>

Primary Cohort (UCSF)

In order to use real-world longitudinal patient data to build and evaluate our models, we utilized the resources made available by the UCSF Clinical Data Research Consultations Team. Each day the team extracts the EHR data from Epic Chronicles into the Epic Clarity relational database (RDB). A subset of that RDB is used to update the Epic Caboodle data warehouse. The Caboodle data is then de-identified using the Safe Harbor method: Private information such as names and addresses are removed. Key personal identifiers are replaced with randomly assigned surrogate identifiers. Dates are shifted by a random number of days from 0 to 364 so that the true date is known no more precisely than the year, however date shifts are kept consistent for each patient so that their chronology is accurately maintained. Ages are adjusted so that patients > 90 years old are presented as 90 years old. Once a month, the de-identified data is extracted to a set of delimited flat files, which our group stands back up into a SQL database. This database contains longitudinal information on over 900,000 individual patients dating from January 2014 to the present.

Inclusion Criteria

Of the over 900,000 patients in the UCSF EHR, 3959 had at least 1 RA diagnosis but only 2452 had 2 diagnoses separated by more than 30 days. Of those 2452 patients, 1417 had been seen by a Rheumatology provider, and only 925 had received at least one CDAI score and only 672 of those patients had received 2 scores. Six hundred and three patients had received at least one inflammatory test and only 578 of the remaining group had also received a DMARD.

© 2019 Norgeot B et al. *JAMA Network Open.*

Replication Cohort (ZSFG)

The IRB for ZSFG did not require de-identification of patient records. EHR data were directly accessed using the eCW product "eBO reports" which runs on an IBM Cognos platform.

Variables Utilized in Model

Medication names were standardized by first using the R scripting library MetaMap [1] and then we programmatically removed any remaining characters associated with delivery or dosage. Medication names were then mapped to the list of DMARDs. Steroids were included if their pharmaceutical class was labeled as "glucocorticosteroid" in the EHR and their route of administration was either oral or injection. All patient medications that did not map to either a DMARD or steroid were dropped. Most machine learning libraries, including the TensorFlow [2] library that we planned to use for modeling, do not accept string values within tensors. Therefore, we encoded medications using a dictionary mapping the drug name to a unique integer value (e.g., Methotrexate= 1) in each patient's record. We chose to include only the first occurrence of each medication given the lack of reliable medication stop dates in the EHR.

Modeling Input Formats

We considered two different formats for representing a patient's longitudinal trajectory as input for modeling. The first method was a Sequential string of events. In this format, each patient's events follow the exact chronology in which they appear within the EHR. As an analogy, in this format, a patient's trajectory is like a sentence and the goal of the model is to predict the final word the sentence (always a CDAI score category in this case of either controlled or uncontrolled). The potential advantages to this format are based on its flexibility: A patient's

© 2019 Norgeot B et al. *JAMA Network Open.*

trajectory is presented to the model in the exact order that it occurred in the hospital, each patient can have an arbitrary number of types of variables, time between patient events can be modeled with fidelity, differences in the numbers, types, and order of patient events can be easily represented for each individual patient. The potential disadvantage of this format is also related to its flexibility: different patients will have different numbers of events and therefore potentially drastic differences in the length of their trajectories which necessitates a more complex model capable of handling the longest and most complicated sequence. From a back-propagation perspective, longer sequences fed into an RNN increase the likelihood of encountering a vanishing gradient thus hindering the model's ability to learn. Within this format the order of variables cannot be anticipated for each patient. Therefore, we prepended the raw values for each variable with a string containing the name of the variable. For example, a raw CDAI score of 10 was converted into ‘cdai10’. Each unique string was then mapped to a unique index. To address the loss of ‘nearness’ introduced by converting continuous variables into uncorrelated strings, we added an embedding layer to all Sequential architectures. Ideally, this would not only allow the network to learn that cdai10 was more similar to cdai11 than cdai50, but also that low CDAI scores were more similar to high ESR or CRP values that appear in close longitudinal proximity for patients.

<u>Example Sequential format:</u>

- Conceptual: (event1_type_value, dT1, event2_type_value, dT2, eventn_type_value)
- Using Real Variables: (ESR11, CRP22, ESR24, methotrexate, CDAI21, PROM50)

© 2019 Norgeot B et al. *JAMA Network Open.*

The second input format that we explored was to force our data to conform to sliding time windows of a fixed interval as is done with most Time Series forecasting. In this format, a window interval is decided upon (for example, 3 months) and variables are decided (for example CDAI, Steroids, CRP). In each window, a single value is entered for each variable. This format is almost universally employed for fields in which variable recording can be guaranteed to be consistent at specific time intervals, such as the stock market or EKG measurements. Outpatient care is by nature inconsistent in the frequency of a patients visits to their care provider and the number of variables that are measured for the patient at each visit. This inconsistency is the result of a combination in patient adherence, the individual and cyclic nature of chronic disease and its severity, and provider preference regarding the types and frequency of measuring patient variables and changing treatment strategies. When the sampling frequency and variable measurement is inconsistent, such as for patients with RA (chronic disease), strategies must be employed to deal with the cases of either having no history of a variable within a given window or having more than one value present for a variable within a single window. Missing categorical variable values such as medications, steroids, and demographics were assigned a one-hot encoded value indicating that the value was not present. There is no reasonable imputation method for the continuous variables in this dataset since they can change drastically over time within a person and have only modest correlation to each other at best. We believe that attempts at imputation are likely to induce bias and spurious correlations, injecting noise into the signal, especially when considering our the relatively small size of our training cohort. We analyzed the continuous variables such as CDAI, ESR, and CRP and determined that a value of zero was never present in the observed data for any of the variables. The field of deep learning has learned empirically that neural networks learn to ignore zero-values assuming that do not legitimately

© 2019 Norgeot B et al. *JAMA Network Open.*

occur as real input values, which is true for our data. While imperfect, we decided that missing continuous variable values would be replaced with zero to indicate that the value was not available. Since our goal was to predict the most recent event, if multiple values occurred for a given variable within a single time window, we selected the most recent value. If a patient's clinical history was too short to fill all windows for a particular experiment, all values for that patient's window were replaced with zeros.

Longitudinal Modeling using Deep Learning

Since patients and their outcomes change over time, and deep RNNs have previously demonstrated superior performance to traditional machine learning methods for chronological EHR data [3], we focused on deep learning models but experimented with many different possible ways to represent time dependencies.

Unlike Random Forests, Support Vector Machines, or Linear Regression, there is no discrete Deep Learning algorithm. Deep Learning is a term used to describe machine learning models based on neural networks of multiple layers and algorithms for their optimization such as stochastic gradient decent and its derivatives. Each layer is composed of a varying number of nodes. The manner in which the nodes operate and are connected to nodes in other layers determines the how the layer transforms the input it receives into the output that it generates. Deep Learning can be viewed as a hierarchical transformation of the input data, each layer acting as a distinct function changing the data in a different way, into the representation of the original input data that makes the predictive task as straightforward as possible. An architecture is an arrangement of layers placed together and representing either the modeler's theory or an

experimental finding about which functions will generate the best representation of the input for a given problem.

Overview of Relevant Deep Learning Layer Types

We considered 5 potential layer types to represent patient longitudinal trajectories: Dense, Time-Distributed, Convolutional, and Recurrent (LSTM, GRU). Each of these layers have distinct methods for generating representations of their input data. Dense layers have no representation of chronology or proximity. Therefore, neither the order of patient events nor which events happened close in time for a patient can be represented. In this way, models composed entirely of dense layers, known as Multilayer Perceptrons, are conceptually similar to Random Forests or kernelized regression. Time-Distributed layers learn a single dense mapping function that is applied to every timestep of the input data. In our case, this can be thought of as a generating one representation for each encounter or window. Convolutional layers can represent proximity but not chronology. Therefore, the representations that they generate can account for which events happened close in time for a patient, but not the global order of events for a patient's trajectory. Models composed of Convolutional blocks, known as Convolutional Neural Networks, offer a distinct advantage in that they use a local pooling of proximity values to reduce the size of the input space, thus reducing the complexity of the model and in-theory increasing its ability to generalize. Despite lacking an explicit ability to directly represent the order of sequences, CNNs have shown to work very well as language models [4], including extracting information from clinical text [5]. LSTMs and GRUs are different variations of Recurrent Neural Networks (RNN), which are the family of architectures that explicitly model chronological sequences of events. LSTMs and GRUs have slightly different mechanisms for learning sequential representations

© 2019 Norgeot B et al. *JAMA Network Open.*

which can lead to differences in performance on different data sets. In general, LSTMs are more robust but due to that they are slower to train.

Model Training

The UCSF cohort was divided into three sub-cohorts for model building and testing: training, validation, and testing. To ensure that the sub-cohorts were representative of the overall population, we calculated the proportion of patients in each CDAI outcome category (60% were Controlled, 40% were Uncontrolled). We then performed a Stratified Random Split, keeping 20% (n = 116) of the patients aside for testing (these patients' data were never trained on, the data was used only to test the final model) and using 80% for model training and development. We then performed an additional stratified random split on the patients assigned, allocating 80% for direct model training (n = 369) and the remaining 20% (n=93) for model validation. This validation cohort was used to assess the generalizability during the model selection process (by varying model architectures and hyperparameters). All continuous variables (CDAI, ESR, CRP) were then linearly scaled to range between zero and one with min/max scaling using the minimum and maximum values for each of these variables found in the training cohort. Thus, the training data were used for model optimization and the validation data were used to guard against overfitting during model selection and hyperparameter tuning. Once a final model was produced, we combined the validation cohort with the training cohort to train the model on both cohorts before the final single evaluation on the test cohort.

The ZSFG patient cohort was less than half the size of the UCSF cohort and we chose not to involve it in model selection. The ZSFG cohort was split in two, the test cohort was matched to

© 2019 Norgeot B et al. *JAMA Network Open.*

the size of the UCSF test cohort as closely as possible (n=117) so that model performance could be evaluated across equally sized patient populations. A training cohort, comprised of the remaining patients (n = 125), was created from the remaining patients. Membership in the cohorts was assigned through a random stratified split as described above.

The goal of any deep learning architecture is to learn a representation of the original input data that maximizes the success rate for the predictive task. In this case, the input data is each individual patients' clinical RA trajectory and the task is to predict what each patients' disease activity state will be at their next visit. The final representation that the architecture generates is captured by a vector which represents the patients chronology, which we have called a Patient Trajectory Vector (PTV). During training, the deep learning architecture is used to generate a PTV. The PTV is fed into a logistic classifier which makes a binary prediction (controlled, uncontrolled) of the patient's disease state at the time of their next visit. The difference between the sigmoidal prediction of the outcome and the patient's actual outcome is the error. The error is then back-propagated into the PTV and then into the architecture itself. The parameters for the architecture are then gently updated in directions that would have led to a better PTV representation for that patient resulting in a sigmoidal output closer to the ground truth (zero for Controlled, one for Uncontrolled). These updated transformations are then applied to next training patient sample and evaluated. In practice, models are updated based on the results of batches of samples instead of single samples because batch-training has been shown to produce models that converge faster [6].

© 2019 Norgeot B et al. *JAMA Network Open.*

For models that included both patient variables that changed over time, such as lab values and CDAIs, and static variables that did not change over time, such as demographics, the variables were separated according to whether or not they were time-dependent. These separate inputs for each patient were fed into two independent deep networks; a recurrent network for the time-dependent variables and a purely dense network for static variables. The two network outputs were concatenated to form a final joint representation which was sent through a non-linear layer and passed to the logistic classifier. Back propagation flowed through both networks allowing joint learning of static and time-dependent representations**.**

Model Optimization

There are many different strategies that can be applied for model optimization. The most common methods for optimization include: experienced intuition, grid searches, random searches, or some form of Sequential Model-based Global Optimization (SMBO) techniques. Since, to our knowledge, no model architectures for multivariable time series deep learning to predict future health outcomes for a chronic disease have been published, there was no data to guide intuition for selecting the optimal variables. While grid searches are popular because they are easy to conduct, Bergstra and Bengio [7] have shown that it is more efficient to randomly search through values while employing a method to intelligently narrow the search space than it is to loop over a fixed sets of hyperparameter values in a grid. SMBOs are algorithms that begin with a random search over the hyperparameter space, and then use the results of the models built with that search to fit one or more surrogate functions that describe the relationship between a set of possible hyperparameters and model generalization. The algorithm then begins optimizing the

© 2019 Norgeot B et al. *JAMA Network Open.*

surrogate function with the goal of identifying points in the hyperparameter space that will lead to improved model performance on data unseen by the model during training.

To account for the slight class imbalance and to further optimize the network two different approaches biasing model predictions towards the different classes of patients (Controlled vs Uncontrolled) were tested. The first was the sampling method used for training. Patient's were sampled for training either randomly or at double the distribution for their class found in data. Additionally, we tested two methods for penalizing the loss function: either equally for each class or proportionally balanced so that the under-represented class was up-weighted.

Model Selection

We favored a model that could explicitly model time, such as deep recurrent model (LSTM or GRU based layer inclusion), so that counter-factual hypotheses could be explored in a manner that would be clinically straightforward (Eg: "What might have happened to this patient if I'd given them Treatment T, 2 visits ago?"). Our goal was to identify the best performing single architecture and hyperparameters, and additionally to uncover trends in performance associated with different ways of representing patient trajectories and the types of patient variables that were most essential for accurate predictions of future patient outcomes. Therefore, we set up separate SMBO experiments for each combination of architecture family: dense, time-distributed, convolutional, and recurrent architecture as well as architectures combining different layer types. Dense Architectures were included primarily because they have been shown to

© 2019 Norgeot B et al. *JAMA Network Open.*

perform equivalently to logistic regression across many different data sets (CITE ME: Logistic regression and artificial neural network classification models: a methodology review, Dreiseitl 2002) and therefore act as a surrogate for logistic regression and a baseline comparator for model selection. If more complex architectures such as LSTMs and exclusively dense networks have comparable AUROC, logistic regression could be considered a sufficient model for this predictive task.

Once the best combination of patient value types and model architecture was identified, additional experiments were performed to determine the impact of the length of patient history to include as input on model performance. Overfitting, the substantial divergence of model log-loss performance between data used for training and unseen data, was rigorously monitored by comparing the model's performance on the training cohort to that of the development cohort. Training for any model was stopped as soon as overfitting was detected. Models were ranked by their AUROC on the UCSF held-out validation cohort, their performance on the training data was never recorded. To eliminate potential complications introduced by multiple hypothesis testing in the space of algorithm comparison, we chose to select only the single model architecture that performed best on the validation cohort to test on the test cohort. We determined to select the architecture with the highest lower-bound Confidence Interval (CI). For example, if architecture X had a CI of [0.7, 0.8] and architecture Y had a CI of [0.6, 0.9], we would select architecture X as the best performing model.

Model training, optimization, and selection were performed using the TensorFlow [2] computational engine wrapped with Keras [8] as a front end on Amazon Web Services Elastic Cloud (EC2) P2XLarge Linux GPU servers. Additional python libraries were used for data

© 2019 Norgeot B et al. *JAMA Network Open.*

preprocessing and model evaluation including Pandas [9], Matplotlib [10], scikit-learn [11], and Numpy [12].

Transfer Learning and Fine Tuning

Transfer Learning is the deep learning practice of taking a model that has been fully trained on one data set and updating the model's parameters by retraining the final dense layer on data from a new data set while keeping the rest of the model parameters frozen. This is generally considered most appropriate when the two datasets are highly similar. For our work, that meant training the model on the UCSF data, then updating the weights for the PTV using the training cohort from ZSFG while keeping the Time-Distributed Dense and GRU layers frozen.

Fine Tuning is a general case of Transfer Learning where layers other than the final dense layer of a model, including potentially all layers, are updated by training on a new data set. This approach is generally most applicable when the two data sets are part of the same general domain but are otherwise very different from each other. We experimented successively unfreezing one additional layer from the top down.

Deep Learning is notorious for requiring training sample sizes far above the number of EHR records for patients with most chronic diseases (even if patients at many large hospitals were aggregated). To overcome this, during our model building process we leveraged physician knowledge and experience to select a small number of raw variables with known clinical importance. This reduced the number of variables the model needed to sift through to learn from, and presumably reduced the number of patient samples necessary to properly train models, by many orders of magnitude. We found that beginning with a small number of clinically important

© 2019 Norgeot B et al. *JAMA Network Open.*

variables, even if the consequences of their complex time-dependent interactions are not perfectly understood, has the added benefit of increasing model interpretability.

The deep neural architecture that performed best was constructed in a way in which a human might approach the problem. The time-distributed layer essentially creates a summary of each time-window for the patient. The recurrent units then look for longitudinal patterns in the chronological summaries from each patient. The dense PTV then generates a single representation of the patient's overall trajectory. It is this complete trajectory representation that the logistic classifier uses to forecast the patient's future CDAI category. The fact that this model also contained the smallest number of trainable parameters and utilized a high degree of regularization also makes sense intuitively, especially considering the relatively small size of the training cohort and the large differences between the two testing cohorts.

eReferences

1. Aronson AR. Effective mapping of biomedical text to the UMLS Metathesaurus: the MetaMap program. *Proc AMIA Symp.* 2001:17-21.
2. Abadi M, Barham P, Chen J, et al. TensorFlow: a system for large-scale machine learning. Proceedings of the 12th USENIX conference on Operating Systems Design and Implementation; 2016; Savannah, GA, USA.
3. Choi E, Schuetz A, Stewart WF, Sun J. Using recurrent neural network models for early detection of heart failure onset. *J Am Med Inform Assoc.* 2017;24(2):361-370.
4. Kim Y. Convolutional neural networks for sentence classification. *arXiv preprint arXiv:14085882.* 2014.
5. Gehrmann S, Dernoncourt F, Li Y, et al. Comparing deep learning and concept extraction based methods for patient phenotyping from clinical narratives. *PLoS One.* 2018;13(2):e0192360.
6. Bottou L. Large-Scale Machine Learning with Stochastic Gradient Descent. 2010; Heidelberg.
7. Bergstra J, Bengio Y. Random search for hyper-parameter optimization. *J Mach Learn Res.* 2012;13:281-305.
8. Chollet F. Keras. In. Vol 1282015.
9. McKinney W. Data structures for statistical computing in python. Paper presented at: Proceedings of the 9th Python in Science Conference2010.
10. Hunter JD. Matplotlib: A 2D Graphics Environment. *Computing in Science & Engineering.* 2007;9(3):90-95.
11. Pedregosa F, Ga, #235, et al. Scikit-learn: Machine Learning in Python. *J Mach Learn Res.* 2011;12:2825-2830.
12. Oliphant TE. *Guide to NumPy.* CreateSpace Independent Publishing Platform; 2015.

© 2019 Norgeot B et al. *JAMA Network Open.*

eFigure 1. Architecture of Best Performing Longitudinal Deep Learning Model

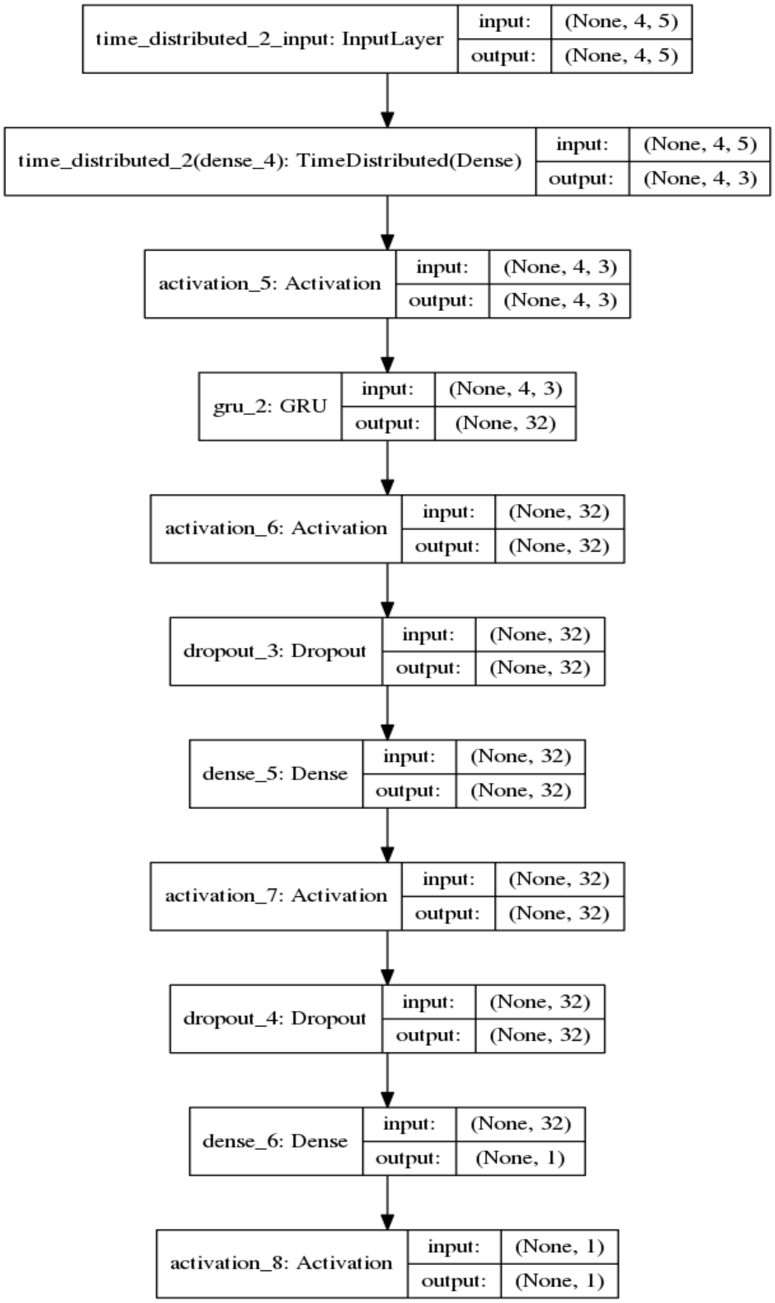

© 2019 Norgeot B et al. *JAMA Network Open.*

eFigure 2. Sensitivity Analysis Comparing Forecasting Performance versus Training Size

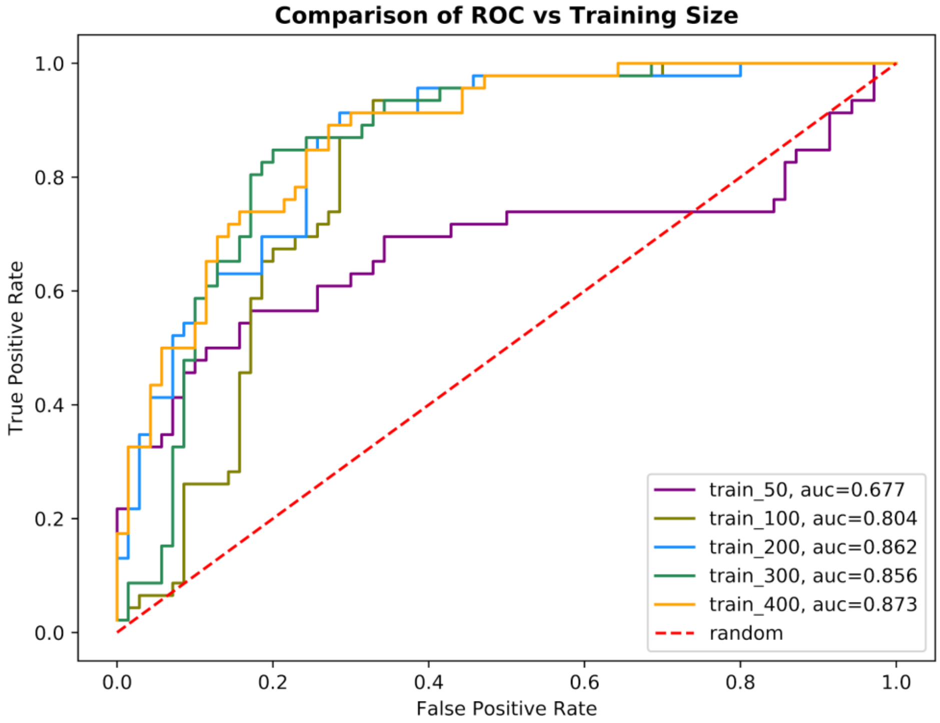

Forecasting performance increases non-linearly with the number of samples available for training. There is a sharp increase in performance between 50 and 100 samples. The net size of performance gains becomes smaller as the sample size is increased. It is important to note that these experiments are conducted post-hyperparameter-optimization. Therefore, they reflect the numbers necessary to train the optimal model but do not reflect the numbers necessary to identify the optimal model.

© 2019 Norgeot B et al. *JAMA Network Open.*
